# Exploring the needs of stroke patients after discharge from rehabilitation centres in Saudi Arabian communities: An IPA qualitative exploratory study design

**DOI:** 10.1371/journal.pone.0291263

**Published:** 2023-09-08

**Authors:** Basema Temehy, Andrew Soundy, Ahmad Sahely, Yasmin Palejwala, Jonathan Heath, Sheeba Rosewilliam

**Affiliations:** School of Sports, Exercise and Rehabilitation Sciences, University of Birmingham, Birmingham, United Kingdom; King Saud University College of Medicine, SAUDI ARABIA

## Abstract

There are about 29 strokes per 100,000 people, annually, in the Kingdom of Saudi Arabia (KSA). These patients require long-term rehabilitation services to enhance recovery and independence in the community. Currently there are limited long-term rehabilitation services in KSA and research is needed to establish pathways for provision of community-based rehabilitation (CBR). To develop effective new CBR models, understanding the experiences and needs of stroke patients in KSA who have undergone poststroke care services is essential. This study aims to gain insight into stroke patients’ needs after their discharge from rehabilitation centres in Saudi Arabia. An interpretive phenomenological analysis (IPA) study was undertaken using semi-structured interviews. Participants were eligible if they had a stroke, completed their in-hospital rehabilitation sessions and had been discharged within the past three years. Semi-structured interviews were conducted using interview guides. Transcripts were translated and analysed using interpretive phenomenological analysis. Twenty-four (15 males and 9 females) participants were recruited from two hospitals in KSA. The key findings suggested that patients experienced limited community rehabilitation services postdischarge unless they were financially able to pay for private therapy. Coping barriers including Medical, Psychological, Social, and Financial and facilitators including Faith, Recovery, Social support and leisure were identified. Participants suggested strategies to improve services within hospital and community for rehabilitation, needs of staff, access to services and ongoing care. Further work is required to develop, implement and evaluate a community rehabilitation intervention that includes education, and self-management elements to support stroke survivors in the community in KSA.

## Introduction

Worldwide, stroke is the third leading cause of death and second leading cause of disability [1]. According to the World Health Organization, stroke is Kingdom of Saudi Arabia’s (KSA) second leading cause of death [2]; the incidence rate of stroke in the KSA is 29 strokes per 100,000 people annually [3]. However, this rate is lower compared with Western countries. In the United Kingdom (UK), there are more than 100,000 strokes annually (162/100000 in 2020/21) [4]. In United States (US), 41.1 per 100,000 died from a stroke in 2021 [5]. Part of the reason for this is because two-thirds of the population in the KSA is under 35 years old [6].

There is a need to provide rehabilitation services to an increasing number of people with impairments following stroke. Evidence suggests that coordinated care for individuals with a stroke can decrease mortality and promote independence [7]. Rehabilitation services in the KSA can be obtained free through the country’s public healthcare system or privately. The Ministry of Health is responsible for providing 60% of healthcare services, and the other 40% is split among other government agencies, such as the Ministry of Defence, the National Guard, universities and the private sector [8]. A multidisciplinary team of physiotherapists, occupational therapists, speech therapists, orthotists and prosthetists are usually included in most stroke rehabilitation programmes in the KSA [9].

Rehabilitation is provided in a hospital over a period of 45 days in the KSA [10]. However, one key limitation in stroke care services in KSA is that 95% of stroke patients receive treatment in nonspecialised stroke hospitals [11]. Only three hospitals, all located in Riyadh, have stroke teams and stroke units [12]. This limited availability of stroke care services in the KSA warrants specialist stroke rehabilitation services involving multidisciplinary team collaboration, stroke units, and specialised stroke training programmes for professionals [13]. More importantly, in the KSA, once a patient completes therapy sessions in an outpatient department, there is limited follow-up rehabilitation in the community. They do not receive the recommended evaluation at six weeks and six months after being discharged from outpatient clinics, nor annual reviews [14]. Hence, they lose the opportunity to have follow-up assessments of their medical, physical, emotional and social requirements, as well as caregiver needs [15]. Hence, patients either seek private sectors to continue rehabilitation or give up rehabilitation because of financial constraints. Ultimately, there is a lack of evidence regarding long-term care approaches in developing countries, including the KSA [16].

To create an effective rehabilitation model for stroke patients extending into the community, the first step is to understand patients’ needs beyond inpatient care [17]. Patients’ unmet needs are the ‘expressed needs not satisfied by current service provision’ [18]. Unmet needs are associated with lower levels of satisfaction with the services and knowledge offered, as well as a lack of knowledge about life after a stroke [19]. Moreover, assessing needs is vital to evaluate the effectiveness of rehabilitation services [20]. Hence, in a context where there are limited follow-up services for patients postdischarge, it is important to explore areas where requirements remain unfulfilled [21].

Exploring unmet needs is a key research priority; for instance, around half of stroke patients in the UK have reported not having their needs met [21], whereas around 96% of people with stroke in Australia reported at least one need not being fully met [22]. Needs should be reviewed periodically to ensure effective intervention and appropriateness of support [23]. Professionals can make the rehabilitation procedure more responsive to patients based on their needs.

Research in Western countries has acknowledged the significance of evaluating patients’ needs [24, 25]. However, patients’ needs in the West is difficult to transfer to the KSA context because of cultural, political and social differences. Further, the focus of previous research has often been narrow; for instance, some studies have solely focused on psycho-emotional needs [26, 27].

Despite the necessity for a needs assessment, to the best of the authors’ knowledge, limited research has explored the experiences and needs of people with a stroke in the KSA. A thorough search of the literature yielded only one prior study evaluating the needs of Saudi Arabian patients undergoing rehabilitation [28]. A survey was utilised to assess the needs of 280 neurological patients in inpatient and outpatient clinics who had experienced stroke (37.1%), spinal cord injury or brain injury. The survey did not acknowledge the needs postdischarge. The survey did not reflect the lived experiences, and the depth and breadth of the research into actual needs were limited. When patients return to the community after hospital-based rehabilitation, challenges arise, with patients still having several requirements that need to be met. In addition, health resources available throughout the rehabilitation process are less accessible, particularly in the KSA, where community-based rehabilitation is lacking [29]. Thus, patients may develop their own adaptation strategies. Identifying these patterns of coping enables practitioners to provide the best possible support to patients. Furthermore, understanding stroke patients’ needs will provide adequate preparation for patients and caregivers to cope in the community. During the transition period from hospital to home, patients and their families need to be prepared for life after discharge via appropriate intervention to reduce associated burdens and prepare them for successful reintegration into the community [17].

To the best of the authors’ knowledge, no research has considered the experience of stroke survivors in Saudi Arabia to explore their unmet needs in a holistic manner, which requires an in-depth data collection method. The present study will be the first step in developing an understanding of patients’ needs and service measures to support patients after discharge, leading to future community-based rehabilitation/support services in the KSA.

The current study aims to identify problems that impact stroke survivors’ adaptation to daily life in the community after discharge from hospital, the factors that influence their adaptation and patients’ needs after their discharge from rehabilitation services.

## Methods

### Study design

The present study adopted an interpretive phenomenological analysis (IPA) qualitative exploratory study design [30]. IPA is a qualitative approach that investigates how individuals make sense of their life experiences [31]. Phenomenology was identified as appropriate since it deals with the interpretation of individual lived experiences. Given the aim of the study and the focus on to exploring patients’ experiences after a stroke. Further consideration to other approaches was not undertaken. For instance, alternative methodologies were not considered like Ethnography focuses on observing shared beliefs and behaviours within a certain culture or Grounded Theory aims to elucidate social processes and develop a theory. IPA is philosophically positioned as a minimal hermeneutic realist [32]. This suggests that an external reality exists, but one must access it through an individual’s personal world. Standards for reporting qualitative research was followed [33]. Ethical approval was provided by two hospitals’ ethics committees, one in the central of KSA, King Fahad Medical City (Institutional Review Board at King Fahad Medical City, Ref: 21-309E) on 10/08/2021, and the other in the south, King Fahad Central Hospital (Jazan Health Ethics Committee, Ref: 2167) on 21/09/2021. Additional review and approval were needed from the University of Birmingham since the first author is a student here (Technology, Engineering and Mathematics (STEM) ethics committee, Ref: ERN_20–1836) on 19/10/2021. Additional information regarding the ethical, cultural, and scientific considerations specific to inclusivity in global research is included in the Supporting Information (S1 Checklist).

### Participants

Participants were recruited if they met the following criteria: (a) were adults (> 18 years of age) who had experienced a stroke (ischaemic strokes and haemorrhagic strokes), (b) had been discharged from the hospitals within the past three years to minimise recall bias and to be relevant to current practice and (c) had completed their rehabilitation sessions in an outpatient clinic. Patients were excluded if they had (a) cognitive disorders (screened using Mini-Mental Status Examination (MMSE) ≥ 24 on zoom [34, 35]), (b) language/communication deficits (identified by a clinician who referred patients to the lead researcher BT) or (c) lived in nursing homes and have paid caregivers who helped with their functional activities.

### Sampling

The study adopted purposive stratified sampling. This non-random sampling method allows researchers to choose individuals with a deep understanding of the issue and who can provide the richest information and insights [36]. Stratification of samples was based on age as it has been identified that patients in working age have different needs compared to patients who are older [37]. Hence two groups were aimed for during data collection. Working age adults was between 18 to 65 years and older patient group was above 65 to 99 years.

### Procedure

Patients who were previously admitted to hospitals were identified based on the eligibility criteria by two rehabilitation specialists from the hospitals’ existing database of patients. Both professionals approached patients who met the inclusion criteria via phone or email to determine interest. The interested individuals’ contact details were passed on to the lead researcher (BT), who is a registered therapist in Saudi Arabia. Individuals who expressed interest received participant information from the lead researcher (BT). The researcher gave them 48 hours to consider participation and offered them an opportunity to ask questions. Individuals who agreed to participate were asked to provide written informed consent in Arabic online. Consents were signed and sent by email or WhatsApp by participants or their relatives.

### Data collection

A semi-structured interview was used [38]. The interviews explored patients’ feelings, knowledge, values and beliefs about their lives postdischarge, starting with simple questions before progressing to in-depth ones.

An interview guide was created based on similar studies [24, 39] (S1 File). The questions covered the interviewee’s life poststroke, support received after discharge, any issues and concerns that reduced their ability to cope, their needs, the type of support offered to manage these needs and any suggestions to improve the quality of the services provided.

Data to define participants’ characteristics such as type of stroke, age, gender, time since stroke, functional ability (Rivermead Mobility Index (RMI) [40]), Barthel Index (BI) [41], Stroke Specific Quality of Life Measure (SSQoL) [42] and MMSE [35] were collected on zoom before conducting the interview.

The interview guide was translated into Arabic by authorised services and was then reviewed and revised by the authors. The questions were tested on one patient who was accompanied by the caregiver and three of the study’s authors via zoom. The cognitive interview was undertaken to test potential issues in the interview questions [43]. The patient was asked to tell us his understanding of the question and to think aloud to analyse his answer and if it was related to the question. Some questions were found to have similar answers, while others were not understandable. Hence, questions were modified following this step. Furthermore, to test the applicability of this schedule, a pilot interview was conducted. The pilot interview demonstrated that it would take over 30 minutes to complete and would likely fall within the current recommendations of between 45 and 60 minutes [44]. Nondirect probing, such as tell me more, keep talking and can you say more about that, was added to obtain in-depth information.

The interviews were in Arabic, which is the mother tongue of both the lead researcher and interviewees. Patients were invited to choose their preferred time and place to undertake the interview, such as face to face in their homes, in the hospital or via phone/zoom. The participants were assured of anonymity and confidentiality to ensure ethical rigour [45]. The study used pseudonyms and removed any identifiable information, such as the participant’s name, profession and organisation, to ensure anonymity and confidentiality. In addition, the subjects were informed of their right to ask questions and to take a break whenever they needed one. The data were recorded using zoom and digital recorders with their permission and transcribed verbatim to minimise the risk of losing data. After each interview, the first author (BT) transcribed the audio tape verbatim. The transcripts were sent to an official translation service to be translated into English. All English transcripts were checked by the lead researcher against the Arabic version to ensure that the translations reflected Arabic meanings; the meaning of the spoken language phrases in the translated transcripts was checked to see if they reflected the original spoken language [46].

### Sample size

The sample size aimed to achieve data saturation and richness of data as is the strategy for qualitative studies [47]. Previous IPA studies found saturation after as few as 12 interviews [48], while others were between 1 and 30 up to 48 individuals [49]. However, in the present study, this occurred at 19 interviews, at which point lead researcher (BT) continued with five more interviews to ensure saturation.

### Analysis

Data were analysed using the IPA analysis tool of qualitative studies [30]. The interviews were read multiple times while listening to audio recordings to create initial notes. The analysis involved writing initial notes about the participants’ sense of person and language used. Exploratory comments involving descriptive, linguistic and conceptual comments were used to create the initial notes. The next step was to code each interview transcript using line-by-line coding about the participants’ context and stance. Then, we established relationships between the clusters of the different interviews one at a time. Specific methods were used to look for patterns and connections between themes [30]. Abstraction is one way in which the cluster is developed by grouping similar codes and then developing a new name. These clusters were added to the main analysis table (master table), which was summarised under themes. The themes were well defined, and the clusters within were checked for appropriateness to fit within these themes. Themes were triangulated between different participants. An example of the coding can be seen in S1 File.

### Rigour and trustworthiness

All interviews were conducted by the lead researcher (BT), a PhD female student at the time of the study and registered physiotherapist in the KSA who had no prior relationship with the interviewees. Investigator triangulation took place: three independent analysts coded five interviews, and their interpretation codes were compared with the main analyst’s coding/interpretation. Once the themes had been developed, the master table was presented to senior researchers for peer examination. The senior researchers refined the wording of the themes and checked for fit between codes and clusters. Furthermore, one interview transcript was checked by a participant (member checking) for accuracy. The logical flow of questions was shown to be adequate, so further interview transcripts were not shared with other participants. A summary of each interview thought/interpretation was made by the researcher. This helped to look for data saturation or additional guiding questions as the data collection progressed. To improve transferability, the context and characteristics of participants are described elaborately. Further, a completed COREQ checklist is attached in S2 File.

## Results

There were 24 participants recruited from two hospitals in the KSA, 10 participants from site 1 (male = 7; Females = 3) and 14 from site 2 (male = 8; Females = 6). The age range of the participants was 29 to 75 years, and the time since their stroke varied from 7 months to 11 years (see details in Table 1). The interviews took place between October 2021 and January 2022 online via zoom. Only one participant expressed willingness towards conducting an in-person meeting; however, this intent could not be carried out due to some constraints, leading to the adoption of an online format for the interview. Other participants expressed a preference for zoom interviews. The interview time ranged from 21 to 49 minutes. Caregivers assisted in the process of arranging the interview if the participants were not able to join. Caregivers were present for 10 interviews and were involved in two interviews. The two caregivers’ data was included in the final analysis.

**Table 1:** Participants’ characteristics.

Participants	Gender	Age	Time post stroke	Type of stroke	Rivermead Mobility Index	Barthel Index	Specific Quality of Life scale	Work
**1**	M	43	1 year	Ischemic	7	90	92	No
**2**	M	53	1 year	Ischemic	2	50	88	Nurse on sick leave
**3**	F	51	1 Y, 2 M	Ischemic	8	70	132	Unemployed after stroke
**4**	F	70	1 year	Ischemic	2	30	126	Unemployed
**5**	M	48	10 years	Not identified	13	65	150	Unemployed
**6**	M	65	9 months	Ischemic	14	95	205	Doctor changed to less intense role
**7**	M	56	1 year	Ischemic	12	75	211	Unemployed after stroke
**8**	M	74	9 months	Ischemic	10	75	173	Unemployed
**9**	F	72	11 years	Haemorrhagic	1	10	89	Unemployed
**10**	F	50	1 year	Ischemic	5	100	134	No
**11**	F	42	3 years	Haemorrhagic	2	20	104	Teacher has asked for retirement
**12**	M	54	2 years	Ischemic	13	100	229	No
**13**	F	64	2 Y, 6 M	Ischemic	13	100	196	No
**14**	M	63	3 years	Hemorrhagic	14	95	221	No
**15**	M	62	7 months	Haemorrhagic	6	80	182	Hospital administration, has retired
**16**	M	65	1 year	ischemic	7	50	143	Retired
**17**	F	59	7 months	ischemic	12	85	153	Unemployed
**18**	M	56	7 months	Ischemic	8	90	143	Private sector, on sick leave
**19**	F	29	8 months	Ischemic	15	100	230	Unemployed
**20**	M	55	1 Y, 2 M	ischemic	3	55	101	Unemployed
**21**	M	62	9 months	Hemorrhagic	11	70	141	Unemployed
**22**	M	41	9 months	Hemorrhagic	11	95	144	Private sector, on sick leave
**23**	M	51	1 year	Ischemic	15	100	237	Private sector
**24**	F	56	1 Y, 6 M	Hemorrhagic	0	0	59	Unemployed

The data revealed three themes: 1) therapy experience, 2) coping poststroke and 3) the need for adequate services. A summary of the themes can be seen in Table 2.

**Table 2:** Summary of the themes and subthemes.

Major theme	Sub-themes	Codes	Definition
**Therapy experience**	Perception of care	Delay diagnosis	This theme illustrates the experience of participants of care with current system.
		Delay in starting physiotherapy	
		Unsatisfied intensity of care	
	Postdischarge	Limited services	The experience that participants had after being released from the facility is described in this theme.
		Loneliness	
	Covid 19 influence	Inability to access services	This theme described how Covid 19 affected on participants care process.
		Progression of condition	
		Seeking private care	
**Coping poststroke.**	Coping barriers	Medical	In this theme, the barriers that affect participants adaptation were descripted including medical, psychological, social, financial and lack of guidance.
		Psychological	
		Social	
		Financial	
		Lack of guidance	
	Coping Facilitators	Faith	This theme descripted the factors that helped participant to adapt poststroke.
		Recovery	
		Social support and leisure	
**Need for adequate services**	Intervention needs	Rehabilitation program	This theme represented the services needed by participants that related to the care programme.
		Staff needs	
		Access to services	
		Hospital needs	
	Care continuity	Ongoing care	This theme demonstrates the services participants require to ensure they can continue therapy after discharge.
		Communication	
		Follow ups	

### Major theme 1: Therapy experience

This theme discusses the experiences of participants regarding the services provided by hospitals from hospitalisation to postdischarge. This theme contains three subthemes: 1) perception of care, 2) postdischarge experience and 3) COVID-19 influence.

#### Subtheme 1: Perception of care

This subtheme illustrates the experience of care participants when within the system. Five participants experienced a wrong or delayed diagnosis and thought that if the diagnosis had been earlier, the long-term disabilities would have been limited. At one site, most participants experienced a delay in starting physiotherapy in outpatients and reported that they waited a long time after discharge from the stroke unit. This made them worry because they thought the delay might hinder their recovery and have a long-term effect on their level of functioning and quality of life. Furthermore, the intensity and frequency of physiotherapy were suggested as being limited in both institutions; patients with moderate and severe strokes wanted intensive therapy. Participant 7 stated, *‘I was undergoing physical therapy sessions at [hospital]*, *but only one session per week*, *as you know*, *one session per week will not work because of my condition … it is not enough*, *the patient needs more time*, *one or two sessions of half an hour each week is not enough’*.

The patients sought physiotherapy input from private clinics or had therapists come home to avoid delaying the start of physiotherapy and increasing the intensity of therapy. Another participant stated, *‘My health condition has improved a lot … but the number of sessions must be increased to get recovered*, *and if the specialist was not at my home*, *my condition would not have improved as required’* (Participant 1).

#### Subtheme 2: Postdischarge experience

The experience that participants had after being discharged from the outpatient facility is described here. Most participants admitted that they had trouble accessing services when they returned to community life; they felt alone postdischarge because there was no support offered and their rehabilitation ended unless they were financially able to access private clinics. One participant stated, *‘They provided me with all the services while I was in the hospital*, *but after I left the hospital*, *there were no longer any services provided to me*, *and I felt alone*, *there is not enough guidance’* (Participant 22).

#### Subtheme 3: COVID-19 influence

The participants were unable to access services during this period. For some, their condition worsened. This was illustrated by participant 13: *‘But in the beginning*, *I did not get treatment because of Corona and the Corona crisis*. *On the day when Corona infections began*, *they closed the outpatient clinic and closed the physiotherapy department*, *so I went there twice*, *and they told me that it was closed’*.

As alternative methods to seek treatment during this period, the participants either sought private therapy or virtual rehabilitation. However, the participants believed that virtual rehabilitation sometimes was not beneficial because they had no experience, no tools and were unable to understand the exercises. Participant 7 stated, *‘They were explaining to me how to do the exercises through the screen; I did not understand the instructions very well*. *And it didn’t work’*. Another said, *‘Sometimes you undergo an exercise on the screen*, *but the tools are not available*, *and sometimes the patient’s application varies’* (Participant 6).

### Major theme 2: Coping poststroke

This theme illustrates the facilitators and barriers and factors that influenced participants’ adaptability poststroke. This theme involves two subthemes: 1) coping barriers and 2) coping facilitators.

#### Subtheme 1: Coping barriers

Several barriers to coping were identified.

- *Medical barriers*. This included issues experienced poststroke, such as physical weakness and inability to do activities of daily living, pain, dizziness, eye issues, sexual issues, sensory issues, shortness of breath, and inability to talk, which limited their adaptation. Participant 22 stated, ‘*My walking is slow*, *and I cannot leave the house*, *and I perform the prayer on the chair … the eyes are one of the sufferings that I complain about*, *I cannot drive*, *I cannot watch TV*, *or read’*.
- *Psychological barriers*. Psychological barriers were identified poststroke, such as depression, an embarrassment of stroke, being a burden on others and the anger of having a stroke. The participants expressed negative emotions such as hopelessness, helplessness, uselessness and frustration with the inability to control things that were no longer under control. Furthermore, the patients described being self-conscious, and they did not want to be pitied. Two participants divorced after having their stroke. One participant illustrated this by stating, *‘I was okay before the stroke*, *and I was married and so happy*. *However*, *today*, *I feel bad*. *Literally*, *I am between life and death waiting for death … unfortunately*, *my wife left me alone’* (Participant 1).
  Furthermore, those participants with a high level of education and who were currently working were more worried about their recovery and trying to return to their previous life. Three participants, including a doctor and teacher, had travelled and spent a substantial amount of money receiving proper rehabilitation. Participant 22 stated, *‘I have one last point*, *which is that from the day I was discharged from the hospital until today*, *there has been no improvement in my condition*, *only very simple improvement … but I will return to work*, *whether I am well or not*. *However*, *I hope my condition improves’*. Another doctor said, *‘I hope to be a surgeon again* … *I no longer have this ability*, *so I’m trying to improve my hand movement’* (Participant 6).
- *Social barriers*. Many people with stroke identified that social participation was limited because of physical weakness, limited transportation, and places not being designed for people with disabilities. For instance, one participant stated, *‘Even if I need something from the market*, *I can’t go get it*, *my sisters help me with that*. *I stay in the car*, *and they get me what I want because there are no special places for wheelchair’* (Participant 10).
- *Financial barriers*. One patient retired because of stroke, three took sick leave from work and had not returned yet, and three were unable to continue working because of physical weakness and had various financial issues. However, most patients’ workplaces were supportive in terms of giving sick leave or changing their responsibilities to what they were able to do. One participant stated, *‘I buy things and then sell them*, *and I have obtained loans from the Jana Foundation to trade*, *but from the month of Rajab until today*, *all my work is suspended*, *and my salary is little*, *and I have an orphaned son and daughter’* (Participant 3).
  Despite this financial pressure, the participants purchased their own walking aids and home modification items to avoid lengthy waits or a lack of response. Participant 15 stated, *‘They told me to log on the internet and fill a form on their website and send it*. *I already sent it*, *but nobody replied’*.
- *Lack of guidance*. The patients suggested that they lacked guidance on what services were available to them, adequate guidance on how to do self-management and what financial supports were available, including aids and home modification support. A few young patients were aware of methods to seek information, such as calling toll numbers and the Ministry of Health. Four participants consulted an expert in the family if they needed guidance, but most waited to ask staff or doctors for follow-up appointments. Sometimes, the internet was used to find information. However, the internet was not a reliable source of information and provided only general information. One participant stated, *‘If I had obtained these special instructions from the hospital directly*, *it would have been better than going to search for this information on the internet because we did not get the appropriate and correct instructions from the internet’* (Participant 23).

#### Subtheme 2: Coping facilitators

There were many facilitators that helped coping.

- *Faith*. Faith that the stroke is a matter of God and that illness is controlled by God helped them cope. The participants also thought that patience and praying to God helped in achieving inner peace. One participant stated, *“When God loves someone*, *he afflicts him*, *and I shall submit to God’s will and be patient and believe in my fate and destiny*, *never to feel sorry for that”* (Participant 1).
- *Recovery*. A better rate of recovery and comparing stroke severity with others helped participants adapt better. One participant stated, *‘There are patients whose condition is worse than mine*, *so I say thank God’* (Participant 7).
- *Social support and leisure*. Social participation and family support, leisure, gardening and reading the Qur’an helped participants improve their psychological status. This theme was illustrated by one participant who stated, *‘My good family dealing with me helped me a lot*, *they always help me to walk*, *they insist for me to walk and go out to the garden or our farm and raise my psychological spirit and they have a great role in psychological support’* (Participant 16).

### Major theme 3: Need for adequate services

Two subthemes under this theme included 1) interventional needs and 2) care continuity.

#### Subtheme 1: Intervention needs

This theme represented the services needed by participants related to their care.

- *Rehabilitation programme*. The patients suggested needs regarding their rehabilitation content: diet advice, speech therapy, psychological rehabilitation, return to work, driving assessment, intensive rehabilitation and providing a short- and long-term treatment plan to keep them informed about their recovery progress. Participant 6 stated, *‘I must receive the necessary treatment through a standardised treatment plan in which all patients are treated equally’*. One caregiver said, *‘I would like the hospital to understand his psychological condition and the degree of frustration he suffers from*. *He does not have any spirit of optimism about tomorrow*. *The psychological state affects the response to treatment; it is half of the entire treatment’* (Caregiver of participant 2). Participant 23 stated, *‘I was hoping that they would put me on a diet programme in the hospital*, *a good diet programme in accordance with my condition because most of those who have had a stroke have a diet programme that must be followed’*.
- *Staff needs*. The participants stated that there were not enough staff in the hospital; thus, they had to rely on trainees with little expertise. Patients preferred to be treated by expert staff. Participant 15 stated, *‘Physiotherapists always bring trainees from university and tell them to check patients because they claim that they are busy’*. One participant said, *‘The number of doctors is few*, *while the number of patients is large*, *so there is no proportion between what is available and what is offered’* (Participant 6). Furthermore, the patients were concerned about staff rotation because they believed that changing their therapist each time wasted their time because the new therapist did not have complete knowledge about their status. Participant 2 said, *‘Physiotherapists change constantly*, *and that’s definitely having a negative impact on the patient’*. Furthermore, at one site, there was no female occupational therapy staff because the female participants wanted female therapists to treat them and not be touched by men for religious reasons. Participant 10 stated, *‘Occupational therapy at the hospital is a men’s section only*, *and I am a woman’*.
- *Access needs*. Not all patients were able to access physiotherapy in their cities because of a lack of physiotherapy departments or because there were no specialised stroke units. Therefore, these patients always sought therapy outside of their cities. Another reason for seeking therapy outside was that they believed therapy in larger cities was more professional and advanced, as evidenced by the fact that the majority of participants had travelled to receive treatment.
  Furthermore, nearly all the participants had not received any postdischarge services apart from private therapy if they were financially able to afford treatment. One participant stated, *‘I went to the Saudi German Private Hospital my family transfer me to them … then I went to Abdulatif Gameel Rehabilitation Centre in Jeddah*, *after that I travelled to Egypt’* (Participant 11). The participants wanted occupational therapy and physiotherapy sessions to be held on the same day. Furthermore, people residing outside of the city need to be scheduled for follow-up visits at several clinics on the same day because travelling for each appointment separately is difficult and expensive.
- *Hospital needs*. *‘The hospital is ill equipped and needs expansion’* was raised by the participants at one site. Furthermore, the aids provided by the hospital were not advanced, and there were no clear methods of aid assessment. The participants were unhappy about the very long waiting times for medical appointments and follow-ups. Participant 12 said, *‘I have suggestions in terms of physical therapy staff*, *expanding the place*, *bringing modern equipment*, *all the devices in the physiotherapy department are old; even medical splints are not suitable’*.

#### Subtheme 2: Care continuity

This theme demonstrates the services that the participants required to ensure they could continue therapy after discharge.

- *Ongoing care*. The patients wanted to extend their treatment in the outpatient department mainly because of the lack of postdischarge services. Furthermore, the participants had difficulty with transport going to the hospital for sessions and needed more home visits. They wanted themselves and their families to be trained in self-management. Those participants who wanted to continue treatment postdischarge either brought in private therapists for sessions at home, performed what the hospital therapist did in the sessions at home or sought out private clinics; however, they viewed factors such as high cost as barriers to continuing treatment in private clinics and barriers to self-management such as the need for motivation, need for training, supervision, confidence and fear of falling. One participant stated, *‘I did not get support or services*, *and as soon as the sessions ended*, *I had no choice but to implement the exercises myself*, *because they did not provide services after the end of the physiotherapy sessions’* (Participant 23). Another participant stated, *‘No*, *I haven’t*. *I have no money to be treated in a private hospital*. *If I had enough money*, *I would go to a private hospital to be treated there’* (Participant 1).
- *Communication*. Patients had difficulty scheduling appointments and were upset because of delays in sessions and the gap between appointments. Furthermore, there was no clear communication channel. Participant 22 stated, *‘But there is no open channel*, *and there is no communication method that enables me to contact directly’*.
- *Follow-up*. The patients wanted continuous follow-up for psychological support. One participant said, *‘No*, *I no longer go to the physical therapy clinic*, *I just refer to the consultant doctor in the neurology clinic’* (Participant 22).

## Discussion

The current study was able to reveal the needs of patients with stroke following discharge from rehabilitation centres, illustrating the participants’ experiences with the current health system, the factors that enabled their coping, facilitators and barriers for recovery, and need for further services.

Stroke survivors in the UK receive around 70 hours of multidisciplinary rehabilitation [50], and a minimum of three hours daily in Australia [51]. Our findings found that the patients in the present study received rehabilitation for two days or less per week. However, most of the study participants claimed that two days per week was insufficient to achieve their target goal of improving physical function. They expressed the need for a more intensive and comprehensive rehabilitation schedule, but staff shortages and heavy workloads were an issue. Current services are insufficient to handle the projected increase in stroke cases because only 5% of strokes in the KSA are admitted to acute stroke units with specialised staff [11]. A survey of staff availability in 2019 showed that there were only 240 physiotherapists working in the KSA at the time, which might be inadequate to care for the rising stroke population [11].

In UK stroke guidelines, all patients should be discharged from the hospital with individualised care plans to provide support by local services to facilitate their reintegration into the community and enhance their quality of life. Furthermore, 95% of stroke survivors are reviewed 12 months postdischarge to assess their needs [52]. However, in practice, nearly half (45%) of patients with stroke in the UK felt unsupported post-discharge from the hospital. Additionally, another 40% are released from the hospital without any further rehabilitation plans [53]. In Australia, one-third of stroke patients are discharged without a care plan [54].

The current study’s patients had limited access to services postdischarge. Community-based rehabilitation services are a necessary component in managing people’s chronic conditions that would enable them to continue to maintain and improve their recovery and help in releasing the pressure on hospitals. However, in developing countries like Saudi Arabia, there are only a few of these publicly funded services, leaving stroke patients limited choices and looking to private healthcare institutions. One effective strategy that can be applied in communities where there is a lack of community-based services is self-management [55]. Self-management training is an effective strategy to meet most needs including a) intensity of care b) long term care continuity c) staff shortage and d) limited post discharge service. Self-management training may be cost-effective in the long term, benefitting both stroke patients’ independence and improving health services [56]. This strategy can ensure that all patients, regardless of their socioeconomic status, receive sufficient treatment. Wang et al. pointed out that mastering self-management skills by acquiring stroke-related knowledge and know-how could lead to positive outcomes [57]; here, setting goals and monitoring progress were found to be the two most common strategies [58]. Professionals need to provide patients and families a clear plan of their rehabilitation. Setting goals in collaboration with the patient, family members and professionals has been suggested as the most advantageous approach [59]. Rehabilitation professionals in the KSA might benefit from using this approach to encourage stroke survivors to pursue their goals.

Another potential approach suggested by a similar community context in Malaysia was to involve family in providing care [60]. Family members who acted as the patient’s caregiver played a significant role in the patient’s successful recovery process [61]. Saudi patients’ psychological well-being was greatly enhanced by the presence of familial social support and was mentioned by 17 participants in the present study. The elderly population of Saudi Arabia would rather obtain care and support from their own families than from other individuals or institutions; it is uncommon for Saudi families to send their elderly relatives to long-term care institutions because it is considered a dishonourable practise. Stroke patients in Saudi Arabia are almost always accompanied by their caregivers when they attend therapy. However, caregivers rarely have the opportunity to understand or acquire the necessary knowledge and skills specific to stroke management and rehabilitation. Further, individuals who have survived a stroke may find it hard to absorb and interpret information and advice, which may explain why some patients have repeatedly asked for information or feel inadequately educated [56]. Many participants in the present study were unable to read (12 participants), so giving instruction and education and training family members, who are acting as caregivers might support rehabilitation efforts. According to Kalra et al., caregiver training not only lowers the cost of care but also greatly enhances the patient’s quality of life [62]. Hafsteinsdottir et al. showed that caregivers should be given training that is targeted, timely, client centre, and tailored to their individual needs and situation. The training should begin at entry time and continue throughout their stay with the patient [63]. Thus, a family-supported treatment could help maintain long-term rehabilitation of stroke patients in the KSA.

For stroke patients to regain optimal health, they need to change and adapt certain health-related behaviours to enhance their recovery [64]. The patients in the current study had limited guidance on various aspects of the recovery process. One solution to meet this need can be in the form of booklets that provide detailed information, as well as answer questions that stroke patients may have about their care, recovery and rehabilitation [65]. However, Rodgers et al. (2001) showed that giving stroke patients and their caregivers booklets was not nearly as effective as enrolling them in an educational programme that gave more in-depth knowledge and know-how of treatment and rehabilitation procedures [66]. These programmes not only raised the quality of the services, but also resulted in better adjusted patients and their families living more satisfactory and functional lives. Furthermore, when information was delivered in a way that actively engaged patients and caregivers, such as by providing multiple opportunities to ask questions, it had a greater impact on patient mood than information delivered on a single occasion [67]. Future predischarge programmes could be introduced into the KSA’s healthcare system, and patients should be adequately and properly equipped before discharge from the hospital into their own homes.

### Limitations

To evaluate the results, some methodological considerations must be taken into account. The study took place in two hospitals, so the findings might not be transferable to other regions. The fact that the study excluded people with severe cognitive impairments and communication disorders is a limitation to the external validity. The findings are less applicable to patients with severe stroke because most had difficulty speaking poststroke. Further, although we aimed to recruit two groups, working age ranging from 18 to 65 years old and older patients from 65 to 99 years old, we recruited only a few patients above 65 because this group was mostly not able to read and did not have contact details, so reaching them was difficult. However, we covered both groups because 11 participants were working while the rest were not. All interviews were not face to face, which may have led to missing signals and clues from facial expression and body gestures [68].

## Conclusion

This phenomenological interpretive study provided an opportunity to explore the needs of Saudi patients with stroke postdischarge. Our findings have indicated a need for greater community access to educational and healthcare resources. These results are crucial in developing and implementing a community intervention to support stroke survivors after discharge. Future qualitative studies need to consider the perceptions and experiences of caregivers because stroke could have different effects on patients and caregivers. This could provide further contributions to creating recommendations for better preparation of stroke survivors supported by their caregivers. Additionally, capturing the changes in individuals’ needs across the care continuum is required to understand the development and meeting of patients’ evolving needs at each stage.

## Supporting information

S1 Checklist Inclusivity in global research. (DOCX)

S1 File Interview questions and example of analysis. (DOCX)

S2 File Consolidated criteria for reporting qualitative studies (COREQ): 32-item checklist. (DOCX)
