# Correction: Exploring the needs of stroke patients after discharge from rehabilitation centres in Saudi Arabian communities: An IPA qualitative exploratory study design

**DOI:** 10.1371/journal.pone.0324195

**Published:** 2025-05-13

**Authors:** Basema Temehy, Andrew Soundy, Ahmad Sahely, Yasmin Palejwala, Jonathan Heath, Sheeba Rosewilliam

An additional affiliation is missing for the first and third author. Basema Temehy and Ahmad Sahely are also affiliated with Physical Therapy Department, College of Nursing and Health Sciences, Jazan University, Jazan, Saudi Arabia.

The corresponding author’s email address is incorrect. The correct email address is: btemehy@jazanu.edu.sa.
