# Supplementary material for: Exploring the needs of stroke patients after discharge from rehabilitation centres in Saudi Arabian communities: An IPA qualitative exploratory study design
Source: PLoS One. 2023 Sep 8;18(9):e0291263. doi: 10.1371/journal.pone.0291263 (PMC10490873; doi:10.1371/journal.pone.0291263)
Supplement: S1 File — (DOCX) [file pone.0291263.s002.docx]

**Exploring the needs of stroke patients after discharge from rehabilitation centres in Saudi Arabian Communities: an IPA qualitative exploratory study design.**

**Basema Temeh****y,* Andrew Soundy^1^, Ahmad Sahely^1^, Yasmin Palejwala^1^, Jonathan Heath^1^ and Sheeba Rosewilliam^1^.**

Interview Questions

1. Can you tell me about your life after the stroke? What aspects have been affected?
2. Can you describe what treatment you have received while you were in the hospital?
3. What were you told about what will happen after discharge from the hospital at that time?
4. What type of support did you receive after discharge? Please explain.
5. Can you describe the support you received from your family? Community?
6. What problems do you think are most important to you after discharge from hospital/rehabilitation centre?
7. What concerns/worries do you have currently? Can you describe them in detail?
8. Please tell me about your needs after stroke that you wish to fulfil?
9. Have you faced any challenges since discharge? If so, what is it and whom do you ask if you need guidance?
10. How do you handle the challenges you face within your daily life?
11. Did you continue rehabilitation in a private clinic? why?
12. What things can help you improve your adaptation to your new life?
13. What are your requirements from rehabilitation/ health services?
14. What kind of support do you think you need in order to manage your current concerns?
15. What do you suggest we can do improve the support we provide for patients who are discharged from health services?

Example of analysis.

| Participant 1 | Interpretation  Coding content, looking for association | Sense of personal and social world; language used and sense of person |
| --- | --- | --- |
| Well, please tell me about your stroke, and what aspects of your life have been affected by the stroke?  First, I had a stroke all of a sudden and I didn't know what happened. But what I want in the end is to get treatment, right? I must receive the necessary treatment through a standardized treatment plan in which all patients are treated equally. For example, when I entered the hospital, there was no neurologist to prescribe treatment for me, but an internist. Do you understand what I mean?  Yes, you mean, there was no specialist doctor!  The operation I underwent was what unsatisfactory.  There was a Saudi surgeon, who was able to enter the place of the clot, I waited for him, and he did not come. There was also a doctor in the hospital, a vascular doctor who is the same as a neurologist.  Aha!  They pointed out to him that if I underwent the operation, I could die, so it was better not to do this operation for me, and I must leave and that I would get better when using the treatment, so I left the hospital with a hemiplegia on the side left, unable to do anything.  Of course, in the beginning, there was no physical therapy for me, it was just a simple movement in the bed, and I underwent a full treatment after that.  How many days did you stay in the hospital inpatient department?  15 days later, I was discharged from the hospital.  What did they tell you at the time of discharge from the hospital?  The decision to leave was made when they noticed that I could eat and drink, be able to answer questions, and could sit in bed unaided.  Well, then I got out of the hospital and started physical therapy right away  Did you start physiotherapy immediately from the first week of your discharge or did you have to wait?  No, I started treatment from the first week of my discharge from the hospital.  Well, what kind of medical rehabilitation did you have in the hospital's physical therapy department?  In the beginning, I could not sit on the bed and could not walk to the bathroom, but after a month of physical therapy, I was able to walk to the bathroom by myself and walk home from room to room, and sit with my family and children well or acceptable and with the continuation of physical therapy my condition improved. Then I entered training clubs (gym), and I improved more than the improvement from physical therapy, my muscles became strong.  How long did you continue to undergo physical therapy? and what did they tell you at the time of discharging the physical therapy department?  A month and a half until I was able to walk. After that I entered or participated in the gym, and going to the weightlifting, I improved a lot and started doing movements that help me how to get up if I fell, so I was trying to learn how to stand myself on my leg. (pause)  I learned from physical therapy how to move, (alright!) I continued to the gym, and I learned how to get up quickly  Was there enough education in the natural department?  There was a good education, yes yes,  despite the lack of hospitals and the lack of doctors, and the physiotherapy centre was far for me, as if I lived in Samtah,  I attended physiotherapy sessions twice a week (Amm). Each session takes half an hour for the movements of the body and hands. I was given five or ten minutes, which was not enough.  My legs improved, but my hands did not.  Although I can move my hands right and left, up and down and move my fingers forward, but it has not improved much. When I try to hold anything light in my hand I do better with difficulty but for heavy things I can do it for five minutes or less, after that the thing falls out of my hand. For example, when I grab a carton from the car home and I can't do it until I reach the door of the house, that thing falls out of my hand.  In the job you work in, were they cooperating with you?  Permeated. I had a four-month long vacation. After that I went back to work, and I was no better tired.  Have you been rehabilitated for returning to work?  (long pause) Yes, I was rehabilitated a little.  Well, how has your social life in general and your family been affected?  My social and family life has been affected to some extent. I was in the hospital helping my colleagues and I could not move my left hand, so I was unable to perform any surgery, so he gave me the clinic to treat patients, examine patients and prepare them for operations, and the staff performs operations for the patients I prepare.  Was there a focus by the physiotherapy clinic on the rehabilitation of delicate functions?  No, there is not. Of course I wanted to be able to walk and move. I didn't want to have an operation or even lift a book or notebook until I was able to walk, so I started thinking about (pause) occupational therapy. But in King Fahd Hospital, there are two doctors who perform occupational therapy, but the number of patients is large. The patient waited until 11:00 pm to receive occupational therapy for 10 minutes. The exercises were not enough, although I do more exercises at home, but it is not enough.  Well, what about the virtual clinic?  not enough  Do you think it is not enough?  Yes. Sometimes you undergo an exercise on the screen, but the tools are not available, and sometimes the patient’s application varies and is not useful unless there is a therapist present with the patient so that he helps him walk and teaches him how to do the exercise correctly.  Well, what was your family's role at home?  My family was helpful, especially my wife, who always encourages me to do the exercises and buy all the required tools.  Were the family trained and instructed on how to help you do the exercises?  My wife, she comes with me in all the sessions, she watches the exercises they give me, and then she applies them at home.  Do you have exercises in place to do at home?  Of course, I got many illustrated exercises, but the patient with a stroke gets tired quickly, especially when moving his hands, he improves drowsiness when he tries to do the exercises, my hands get tired quickly.  What are the things that helped you adapt to the disease?  Going back to work and trying to get back to a normal life may have clearly helped me cope with the disease. Also, the family had a major role in overcoming the crisis. And my wife encourages me that the problem is psychological rather than physical and accept the new situation.  Well, what problems did you face after you left the physiotherapy department after completing all the physiotherapy sessions?  As I told you, occupational therapy has not changed, and I hope to be a surgeon again. I was good doing operations. For example, my colleagues perform about 10-15 operations per month, while I used to perform 80 operations per month. (pause) I no longer have this ability, so I'm trying to improve my hand movement but so far I haven't found a way to do it and I'm still undergoing physiotherapy and occupational therapy at home.  Well, have you completed treatment in private hospitals? and why?  Yes, I visited the Czech Specialist Hospital, and I paid them about 100,000 Saudi riyals, but I never benefited. King Fahd Hospital was better than Czech Specialist Hospital.  I never got better.  But as I told you in King Fahd Hospital, the number of doctors is few, while the number of patients is large, so there is no proportion between what is available and what is offered.  In your opinion, what is the solution to this problem?  There are many problems in the area. The number of physical therapists is large in each region and is sufficient to cover the needs in hospitals. In order for the doctor to train and benefit gradually and gain experience, I sometimes go to private hospitals and there are Saudi doctors who are OK. I mean, they were good, and I benefited from them.  While the Indian doctors have extensive experience and gained experience, these are minor problems, and the hospital is ill-equipped.  Regarding tools such as crutches  Tools are available and I got crutches and splints. But the splints were primitive, meaning not advanced, like those in private hospitals. They had German splints that were comfortable for the hands. For example, they gave me leg splints at King Fahd Hospital, it affected my leg, and I could not walk because of it. Even if I try to walk, I cannot for more than ten minutes and I feel as if I have nails in my leg because these splints are made of plastic, so they are uncomfortable and do not give good results. They are rough and uncomfortable.  Well, at the moment if you have a problem who do you want to ask for help, is there an official way of communication with the specialists?  Do you mean in terms of physical therapy?  Of course.  Yes, they gave me phone numbers that I can call, but after 9 months, I and my children have enough experience to get a response to any answer by searching the Internet and getting solutions and answers to questions, and this is an easy way to get information.  Well, what requirements do you currently need?  (Long pause) of course, psychological problems are the most important component. The patient wishes to return to work, interact with colleagues in the workplace, feel psychological comfort and help recover more. For example, when I go to work in the hospital, I come home happy. I had very simple operations and I felt good about myself, and I was happy and at ease, my family also felt that I was happy much better than any other time. This helps the patient in my opinion to rehabilitate him through society and work.  Well, what would you suggest to improve these aspects? What do you require from medical rehabilitation centers?  First, (pause) the Ministry of Health is supposed to recommend the return of all patients to their work. For example, if we assume that a person has suffered a stroke in the left side of the brain or in the place that controls speech or controls thinking, then it can be facilitated to return to work. If the stroke is on the right side of the brain, this means loss of movement.  So, I recommend the rehabilitation of patients, I mean, I saw people who had a similar stroke, but they improved more because they go for walks on the ground, plow and plant and thus benefit while I am at home, and if I meet a surgeon and help him, then no, I will not restore my skills. I hope patients can be helped back to work.  I had a stroke at ten o'clock suddenly when I was visiting patients who underwent surgery, and my colleague and I were suddenly feeling that I could not stand on my leg and I felt weak. Whoever is injured during work is treated like a Saudi and receives a salary and compensation, but you did not receive compensation and I think that you personally have nothing to do with this.  Thank you for your cooperation with us and  do you have other suggestions or requirements?  No thank you and may God bless you and grant you success.  Thank you so much  **Conversation with the patient's carer:**  A Jordanian doctor with British-German citizenship, I do not know him. I looked at him and noticed that he did not give the patient the thing I wanted, he only gave him a simple thing and gave him more than one session. As for King Fahd Hospital, it only needs expansion and a sufficient number of doctors because the patient needs a session of at least one hour. This is my observation. In terms of performance, they are excellent. I was hoping, Doctor, in his name, that they would open a hospital in Samtah for physiotherapy. The employees receive a salary while they are not working.  I mean, the student works hard and makes the most precious and tries to obtain mediation at any cost in order to get a job, and in the end you have no role and you stay next to your wife at home and take a salary and the sick suffer! And we hear the saying that you are the son of the country first in the job.  So you must do your homework, a year has passed and you are still sitting at home, and you are getting a salary, why!  What about virtual clinics!  Yes, virtual clinics. My husband did not benefit from anything from Samtah Hospital. When the hospital was closed, they came home for ten minutes several times, and then went to home medicine twice.  After that, the Corona virus spread, and the centers were closed, and there was a children's section, and there were no equipment, why? There should be a physical therapy department, where is the head of the department? Why didn't you take any part? You have more than ten employees and this helps reduce pressure on King Fahd Hospital.  Because in fact, Samtah Central Hospital, my husband is a surgeon, they should not have demolished the hospital because it is in a war zone and a confrontation plan, and it receives the wounded and my husband receives cases and therefore they need physiotherapy. But after the center was closed, I brought an Indian doctor home and shoot a video, because my son is studying medicine, so we give him five minutes sessions a day, even if we go to King Fahd Hospital, the sessions are not enough because they say we can complete them at home, and my husband needs three sessions Physiotherapy a day.  We ask you, Doctor, to deliver the message. We want them to open a Samtah hospital, because it will feed the area, fall into a confrontation plan, and be the place of reception for soldiers, and this helps relieve pressure on King Fahd Hospital. | Event identification. No previous knowledge on stroke sign and symptoms.  Seeking treatment  No standardized treatment plan for stroke pts in SA.  Lack of stroke specialists in SA  Unsatisfaction with the operation  Hope to be treated by specialist.  Hope to be treated by specialist.    Problem identification.  Body positioning treatment at inpatient.  Stroke effect on patient life  No problems with long waiting to be admitted  Stroke effect on patient life (unable to do ADL).  Improvement in patient abilities as a consequence of PT  Improvement happened due to PT.  Beliefs on more exercise led to more improvement  .  Patient ability improved with time.  Beliefs on more exercise led to more improvement  Self-learning on stroke recovery  Patient copy what he learnt from PT during his rehabilitation.  Education in outpatient was satisfactory.  Services shortage (hospitals and staff).  Frequency of treatment (twice a week)  Duration of treatment (half hour PT, 10 min OT).  Therapy duration was not enough  Beliefs on intensive treatment. Little therapy means little improvement  Desire to return to normal  Stroke effect  Difficulty in ADL.  Patient did not face problem at work as consequence to stroke  RTW rehabilitation.  Stroke effect on patient life  Work changes due to stroke  No RTW rehabilitation  First goals for patient after stroke  Hope of return to normal life  Staff shortage  Large number of patient  OT duration was not enough  OT duration was not enough  Virtual clinic is existed in SA  Factor of not wanting VC:  Tools were not available  Incorrect application of exercises  Need supervision  Family support  Motivation from family  No official family training in SA.  Need for motivation  Factor helps in adaptation:  Return to work  Family  Family motivation  Accept the situation  OT needs  Hope to return to normal life.  Patient was successful in his job  Stroke effect:  Did not return to normal job  Still searching for ways to recover  Care continuity at home  Care continuity  Private clinic was not beneficial  Public hospital care better than private clinics  Private clinic was not beneficial  Staff shortage in public hospitals comparing to received patients  SA has a lot of issues related to rehabilitation  Staff shortage  Therapists need experience and training  Saudi staff is less experience  Indian staff are expert in PT  Ill equipped hospital.  Availability of tools  Tools are not advanced in public hospitals  Private hospitals provide advanced tools  Tools are not advanced in public hospital  Tools were not comfortable  Phone was given to participant as communication way.  Participant becomes expert with time  Internet is an easy way to get any information  The most important issue for stroke is psychological problem.  Hope to return to normal life (work).  Factors help to recover:  Return to work  Psychological comfort  Doing what participant used to (operation) make him and his family happy.  Rehabilitation needs:  Social participation  RTW  Rehabilitation needs:  RTW  Rehabilitation to RTW  Recovery rates differ  Ways to have high recovery rate:  Working, walking.  No sedentary life styles  Training again on work  Hope to return to normal life (work).  Stroke happened at workplace.  Did not received sufficient rehabilitation  Public hospital needs:  Expansion  Staff  Enough staff helps to have sufficient rehabilitation  Need for closer centre for rehabilitation  upset from closing the nearer centre  upset from Saudi staff  No benefits from VC  Rehabilitation duration was not sufficient  Upset due to closing the near centre  Referral from other hospitals increase the pressure on other hospitals.  Upset due to closing the near centre  Care continuity:  Brought external therapist to home  Family learning to give intensive therapy  Therapy session were not sufficient.  Instruction on home rehabilitation.  Beliefs on more rehabilitation equal more recovery  Need for close centre for care continuity | Stroke awareness  Treatment plan  Specialist staff shortage  Patient is a doctor and knows staff in the hospital  Patient regret the doctor choice of not doing the operation  due to patient condition (unable to move)  Patient liked his improvement to continues PT  Patient received little PT comparing to self-treatment, therefore he sought gym  Apart from his satisfaction, patient was a doctor and able to locate the information himself  Distance problems (the centre close to the patient home is closed due to maintenance.  That why patient sought gym training.  Patient is a doctor and he used to do operation which require fine movement.  Probably desire to return to normal work life  Although patient said he received RTW rehabilitation.  Patient repeated that he was receiving only 10 min OT  Home exercise was not enough? Probably patient wanted to receive OT under supervision.  Why patient did not want Virtual rehab?  Patient’s wife were insisted for her husband to return to his normal life and work.  Patients’ carer watch the exercises performed by PT and copy it. Sometimes even without explanation why we are doing this exercise  Patient is living in SA due to his job, if he lost the job he will lose the benefits of being a doctor.  Patient feel sorry about himself and keep trying to return to his life.  Looking for intensive care to return to his life  Why patient is saying Czech Specialist Hospital is not beneficial although it is specialist stroke care?  Regret paying 100,000  Want to return to normal life in short period.  Meaning there are a lot of therapists have no job can cover the shortage I the city.  Therapist gave their phone number to patient if they asked but there are no official communication service unless he/she go to the hospital.  Participant feel sorry about himself as he was so successful in his job and suddenly lose that.  Carer is talking about Czech Specialist Hospital as she regret going there and paying 100,000 for nothing  The centre is closed for maintenance and clinics turned to virtual clinics or home visits. However, the carer is upset due to this |

Example of author 2 analysis

| **Transcript** | **Codes** | **Themes** |
| --- | --- | --- |
| Are you ready?  Yes, I am  Well, please tell me about your stroke, and what aspects of your life have been affected by the stroke?  First, I had a stroke all of a sudden and I didn't know what happened. But what I want in the end is to get treatment, right? I must receive the necessary treatment through a standardized treatment plan in which all patients are treated equally. For example, when I entered the hospital, there was no neurologist to prescribe treatment for me, but an internist. Do you understand what I mean?  There was no specialist doctors!  The operation I underwent was what unsatisfactory (upset).  There was a Saudi surgeon, who was able to enter the place of the clot, I waited for him, and he did not come. There was also a doctor in the hospital, a vascular doctor who is the same as a neurologist.  Aha!  They pointed out to him that if I underwent the operation, I could die, so it was better not to do this operation for me, and I must leave and that I would get better when using the treatment, so I left the hospital with a hemiplegia on the side left, unable to do anything.  Of course, in the beginning, there was no physical therapy for me, it was just a simple movement in the bed, and I underwent a full treatment after that.  How many days did you stay in the hospital inpatient department?  15 days later, I was discharged from the hospital.  What did they tell you at the time of discharge from the hospital?  The decision to leave was made when they noticed that I could eat and drink, be able to answer questions, and could sit in bed unaided.  Well, then I got out of the hospital and started physical therapy right away, yes  Did you start physiotherapy immediately from the first week of your discharge or did you have to wait?  No, I started treatment from the first week of my discharge from the hospital.  Well, what kind of medical rehabilitation did you have in the hospital's physical therapy department?  In the beginning, I could not sit on the bed and could not walk to the bathroom, but after a month of physical therapy, I was able to walk to the bathroom by myself and walk home from room to room, and sit with my family and children well or acceptable and with the continuation of physical therapy my condition improved. Then I entered training clubs, and I improved more than the improvement from physical therapy, my muscles became strong.  How long did you continue to undergo physical therapy? and what did they tell you at the time of discharging the physical therapy department?  A month and a half until I was able to walk. After that I entered or participated in the gym, and going to the weightlifting in gym, I improved a lot and started doing movements that help me how to get up if I fell, so I was trying to learn how to stand myself on my leg. (pause)  I learned from physical therapy how to move, (alright!) I continued to the gym, and I learned how to get up quickly  Was there enough education in the natural department?  There was a good education, yes yes, despite the lack of hospitals and the lack of doctors, and the physiotherapy centre was far for me, as if I lived in Samtah, I attended physiotherapy sessions twice a week (Amm). Each session takes half an hour for the movements of the body. And hands I was given five or ten minutes, which was not enough. My legs improved, but my hands did not. Although I can move my hands right and left, up and down and move my fingers forward, but it has not improved much. When I try to hold anything light in my hand I do better with difficulty but for heavy things I can do it for five minutes or less, after that the thing falls out of my hand. For example, when I grab a carton from the car to home and I can't do it until I reach the door of the house, that thing falls out of my hand.  In the job you work in, were they cooperating with you?  Permeated. I had a four month long vacation. After that I went back to work and I was no better tired.  Have you been rehabilitated for returning to work?  (long pause) Yes, I was rehabilitated a little.  Well, how has your social life in general and your family been affected?  My social and family life has been affected to some extent. I was in the hospital helping my colleagues and I could not move my left hand, so I was unable to perform any surgery, so he gave me the clinic to treat patients, examine patients and prepare them for operations, and the staff performs operations for the patients I prepare.  Was there a focus by the physiotherapy clinic on the rehabilitation of delicate functions?  No, there is not. Of course I wanted to be able to walk and move. I didn't want to have an operation or even lift a book or notebook until I was able to walk, so I started thinking about (pause) occupational therapy. But in King Fahd Hospital, there are two doctors who perform occupational therapy, but the number of patients is large. The patient waited until 11:00 pm to receive occupational therapy for 10 minutes. The exercises were not enough, although I do more exercises at home, but it is not enough.  Well, what about the virtual clinic?  not enough  Do you think it is not enough?  Yes. Sometimes you undergo an exercise on the screen, but the tools are not available, and sometimes the patient’s application varies and is not useful unless there is a therapist present with the patient so that he helps him walk and teaches him how to do the exercise correctly.  Well, what was your family's role at home?  My family was helpful, especially my wife, who always encourages me to do the exercises and buy all the required tools.  Were the family trained and instructed on how to help you do the exercises?  My wife, she comes with me in all the sessions, she watches the exercises they give me, and then she applies them at home.  Did you receive exercises booklet to do at home?  Of course, I got many illustrated exercises, but the patient with a stroke gets tired quickly, especially when moving his hands, he improves drowsiness when he tries to do the exercises, my hands get tired quickly.  What are the things that helped you adapt to the disease?  Going back to work and trying to get back to a normal life may have clearly helped me cope with the disease. Also, the family had a major role in overcoming the crisis. And my wife encourages me that the problem is psychological rather than physical and accept the new situation.  Well, what problems did you face after you left the physiotherapy department after completing all the physiotherapy sessions?  As I told you, occupational therapy has not changed, and I hope to be a surgeon again. I was good doing operations. For example, my colleagues perform about 10-15 operations per month, while I used to perform 80 operations per month. (pause) I no longer have this ability, so I'm trying to improve my hand movement but so far I haven't found a way to do it and I'm still undergoing physiotherapy and occupational therapy at home.  Well, have you completed treatment in private hospitals? and why?  Yes, I visited the Czech Specialist Hospital, and I paid them about 100,000 Saudi riyals, but I never benefited. King Fahd Hospital was better than Czech Specialist Hospital.  I never got better.  But as I told you in King Fahd Hospital, the number of doctors is few, while the number of patients is large, so there is no proportion between what is available and what is offered.  In your opinion, what is the solution to this problem?  There are many problems in the area. The number of physical therapists is large in each region and is sufficient to cover the needs in hospitals. In order for the doctor to train and benefit gradually and gain experience, I sometimes go to private hospitals and there are Saudi doctors who are OK. I mean, they were good, and I benefited from them.  While the Indian doctors have extensive experience and gained experience, these are minor problems, and the hospital is ill-equipped.  Regarding tools such as crutches!  Tools are available and I got crutches and splints. But the splints were primitive, meaning not advanced, like those in private hospitals. They had German splints that were comfortable for the hands. For example, they gave me leg splints at King Fahd Hospital, it affected my leg, and I could not walk because of it. Even if I try to walk, I cannot for more than ten minutes and I feel as if I have nails in my leg because these splints are made of plastic, so they are uncomfortable and do not give good results. They are rough and uncomfortable.  Well, at the moment if you have a problem who do you want to ask for help, is there an official way of communication with the specialists?  Do you mean in terms of physical therapy?  Yes  Yes, they gave me phone numbers that I can call, but after 9 months, I and my children have enough experience to get a response to any answer by searching the Internet and getting solutions and answers to questions, and this is an easy way to get information.  Well, what requirements do you currently need?  (Long pause) of course, psychological problems are the most important component. The patient wishes to return to work, interact with colleagues in the workplace, feel psychological comfort and help recover more. For example, when I go to work in the hospital, I come home happy. I had very simple operations and I felt good about myself, and I was happy and at ease, my family also felt that I was happy much better than any other time. This helps the patient in my opinion to rehabilitate him through society and work.  Well, what would you suggest to improve these aspects? What do you require from medical rehabilitation centers?  First, (pause) the Ministry of Health is supposed to recommend the return of all patients to their work. For example, if we assume that a person has suffered a stroke in the left side of the brain or in the place that controls speech or controls thinking, then it can be facilitated to return to work. If the stroke is on the right side of the brain, this means loss of movement.  So I recommend the rehabilitation of patients, I mean, I saw people who had a similar stroke, but they improved more because they go for walks on the ground, plow and plant and thus benefit while I am at home, and if I meet a surgeon and help him, then no, I will not restore my skills. I hope patients can be helped back to work.  I had a stroke at ten o'clock suddenly when I was visiting patients who underwent surgery, and my colleague and I were suddenly feeling that I could not stand on my leg and I felt weak. Whoever is injured during work is treated like a Saudi and receives a salary and compensation, but you did not receive compensation and I think that you personally have nothing to do with this.  Thank you for your cooperation with us do you have other suggestions or requirements?  No thank you and may God bless you and grant you success.  Thank you so much  **Conversation with the patient's carer:**  A Jordanian doctor with British-German citizenship, I do not know him. I looked at him and noticed that he did not give the patient the thing I wanted, he only gave him a simple thing and gave him more than one session. As for King Fahd Hospital, it only needs expansion and a sufficient number of doctors because the patient needs a session of at least one hour. This is my observation. In terms of performance, they are excellent. I was hoping, Doctor, in his name, that they would open a hospital in Samtah for physiotherapy. The employees receive a salary while they are not working.  I mean, the student works hard and makes the most precious and tries to obtain mediation at any cost in order to get a job, and in the end you have no role and you stay next to your wife at home and take a salary and the sick suffer! And we hear the saying that you are the son of the country first in the job.  So you must do your homework, a year has passed and you are still sitting at home, and you are getting a salary, why!  What about virtual clinics!  Yes, virtual clinics. My husband did not benefit from anything from Samtah Hospital. When the hospital was closed, they came home for ten minutes several times, and then went to home medicine twice.  After that, the Corona virus spread, and the centers were closed, and there was a children's section, and there were no equipment, why? There should be a physical therapy department, where is the head of the department? Why didn't you take any part? You have more than ten employees and this helps reduce pressure on King Fahd Hospital.  Because in fact, Samtah Central Hospital, my husband is a surgeon, they should not have demolished the hospital because it is in a war zone and a confrontation plan, and it receives the wounded and my husband receives cases and therefore they need physiotherapy. But after the center was closed, I brought an Indian doctor home and shoot a video, because my son is studying medicine, so we give him five minutes sessions a day, even if we go to King Fahd Hospital, as the sessions are not enough because they say we can complete them at home, and my husband needs three sessions Physiotherapy a day.  We ask you, Doctor, to deliver the message. We want them to open a Samtah hospital, because it will feed the area, fall into a confrontation plan, and be the place of reception for soldiers, and this helps relieve pressure on King Fahd Hospital. | Lack of speialists staff  No physio at the beginning  Happy with physiotherapy care  Going to gym  Motivation is essential for self-management  Patient education  Therapy wasn’t enough  Impact of stroke on family and social life  Impact of stroke on job    Patients’ need of rehabilitation weren’t met/motivation is essential for recovery  Poor resources/staff shortage  Not prepared/not equipped for remote rehabilitation  Virtual rehabilitation is useless  Family support is important  Family engagement helps to improving recovery  Providing information  Reintegration into normal life help coping with difficulties  Slow recovery of UL  Private care is costly and not beneficial  Public health doesn’t provide advance equipment  The internet provides helpful information  The need for psychological support  The need for patient reintegration in the community  The need for goal setting/ Back to work is a primary goal of rehab  Staff shortage/building capacity  The therapy wasn’t enough  The rehabilitation completely stopped during COVID  Carers advocate for reopening of closed rehab centers in their area | Unsatisfaction with the quality of care  Lack of support  Patients’ satisfaction with care  Resources of support  Self-management  Self-management  Unsatisfaction with the quality of care  Challenges after stroke  Unsatisfaction with the quality of care/self-management  Unsatisfaction with the quality of care  Challenges  Resources of support    Resources of support  Self-management  Resources of support  Challenges  Challenges  Unsatisfaction with the quality of care  Resources of support  Patients’ needs  Patients’ needs  Unsatisfaction with the quality of care/ patients’ concerns  Unsatisfaction with the quality of care  Challenges  Patients’ needs |
